# Fast Fragmentation of Networks Using Module-Based Attacks

**DOI:** 10.1371/journal.pone.0142824

**Published:** 2015-11-16

**Authors:** Bruno Requião da Cunha, Juan Carlos González-Avella, Sebastián Gonçalves

**Affiliations:** 1 Instituto de Física, Universidade Federal do Rio Grande do Sul, Porto Alegre, RS, Brazil; 2 Departamento de Polícia Federal, Porto Alegre, Brazil; 3 Departmento de Física, Pontificia Universidade Católica, Rio de Janeiro, RJ, Brazil; University of Jaén, SPAIN

## Abstract

In the multidisciplinary field of Network Science, optimization of procedures for efficiently breaking complex networks is attracting much attention from a practical point of view. In this contribution, we present a module-based method to efficiently fragment complex networks. The procedure firstly identifies topological communities through which the network can be represented using a well established heuristic algorithm of community finding. Then only the nodes that participate of inter-community links are removed in descending order of their betweenness centrality. We illustrate the method by applying it to a variety of examples in the social, infrastructure, and biological fields. It is shown that the module-based approach always outperforms targeted attacks to vertices based on node degree or betweenness centrality rankings, with gains in efficiency strongly related to the modularity of the network. Remarkably, in the US power grid case, by deleting 3% of the nodes, the proposed method breaks the original network in fragments which are twenty times smaller in size than the fragments left by betweenness-based attack.

## Introduction

Network theory and its applications pervade many scientific fields, like physics, sociology, engineering, epidemiology, biology, and many others. In this context, three important concepts have received much attention recently: interdependent graphs [1, 2], communities (or modules) [3–8], and robustness of networks facing targeted attacks [9–12]. In the present work we address and bring together these last two concepts.

The resilience of networks against failures or targeted attacks to its components and the subsequent impact of these attacks on the performance of the system have become important practical issues in the last years [13–18]. The robustness of a network is generally associated to the structural functionality of the system, so it is directly related to the fraction of vertices or edges that should be removed in order to stop the network from functioning as a whole —for example when information cannot propagate over the entire network. For instance, the failure of Internet routers [19, 20], the vaccination of individuals to prevent the spread of a disease [21, 22], and the fight against organized crime and terrorist groups [23, 24] can all be described by procedures in which a certain number of vertices in the network is removed. In terms of the attacking procedure, the challenge is to find a list of vertices or edges whose removal would cause high damage to the network. On the other hand, if the aim is to protect a network from attacks, knowing such a list would help to devise an efficient strategy of defense. Hence, the question we want to address in this contribution is: How to cause the same damage as the one resulting of a traditional centrality-based attack on a given network, but removing a smaller amount of nodes or edges?

Pursuing this idea, several centrality indexes have been proposed aimed to measure the structural importance of nodes and edges [25, 26]. For instance, the concept of bridging nodes in the topology of complex networks has been brought to discussion recently [27]. Hwang *et al*. [28] define a bridging centrality in order to characterize the location of central nodes among vertices with high degree. The method succeeds in identifying functional modules but does not show significantly better results than betweenness-based attacks when it comes to atomize complex networks. Marcus and Hilgetag [29] speculated that connections between clusters might be generally important for predicting vulnerability and that their position can be identified using the edge frequency measure (*i.e*. betweenness centrality). Later, Bu *et al*. [30] have studied how the removal of bridging edges affects the epidemics size, but with focus on local strategies with limited knowledge of the network topology. Broadly speaking, these last contributions identify the nodes connecting distinct communities as the ones with high betweenness centrality. Besides, those works were published before the widespread availability of topological community detection algorithms and so the authors did not extract communities from the networks in the formal sense generally used today. More recently, Shai *et al*. [31] have studied analytically the vulnerability of modular Erdös-Rényi networks to both random failures and targeted attacks to bridge nodes. Thus, bringing together previous ideas on attacking bridges among communities and recent developments in community extraction algorithms from complex graphs [32, 33] draws a promising pathway in shaping attack strategies. Even though some major advances have been made in the last few years, the effects of community-based attacks on real complex networks is still an open subject. This is precisely the topic we want to address in this contribution.

**Fig 1:**
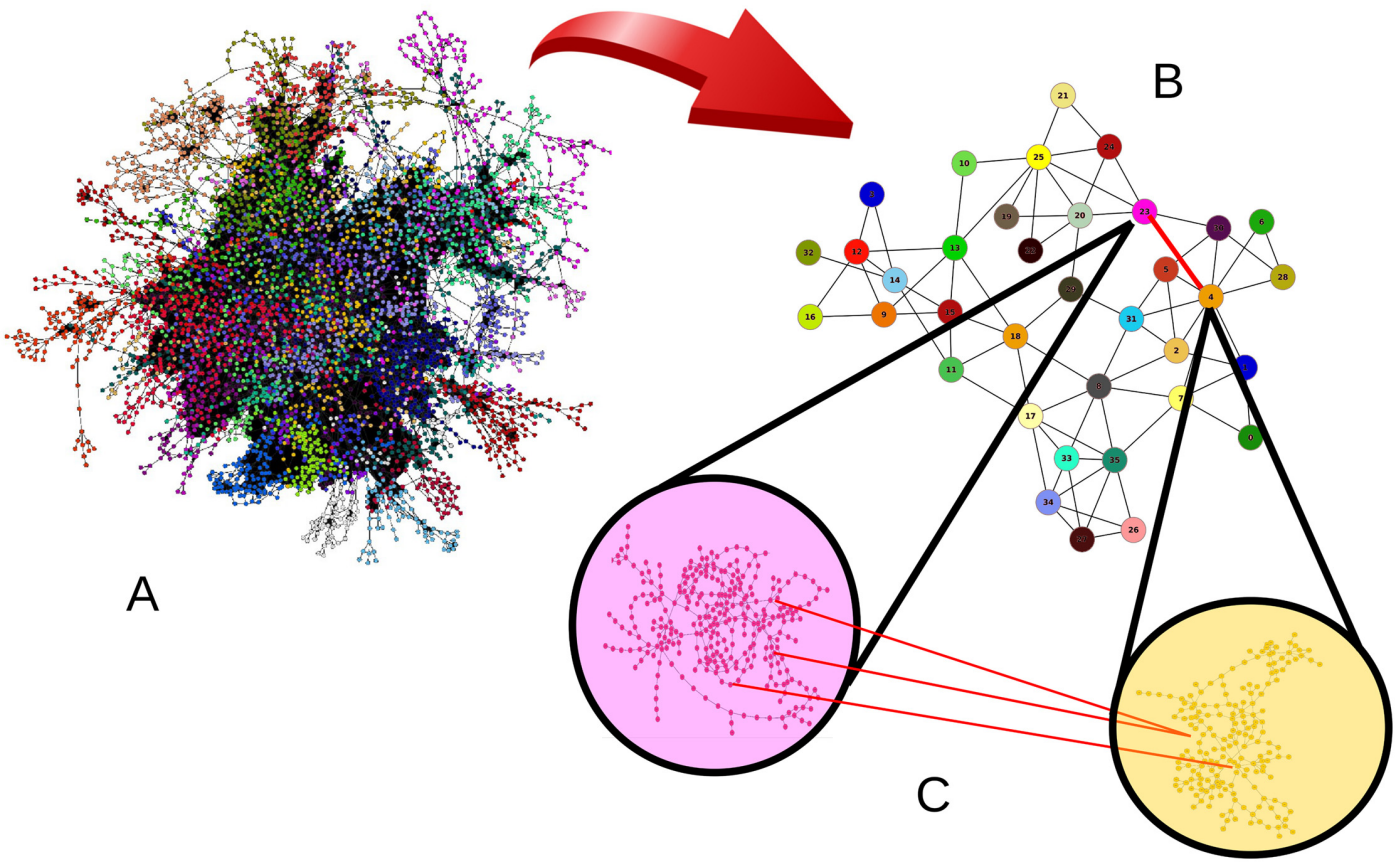
Graph representation of the Western US power grid (A), one possible module representation (B), and the internal structure of nodes and edges inside two selected modules, plus edges connecting nodes between the two modules (C).

In general, communities or modular structures are topological partitions of graphs with dense internal connections but weakly connected among them [34]. In other words, the concentration of links within the modules is greater than the concentration of links connecting them. This structural configuration allows us to identify which are the nodes and edges that connect the modules, *i.e*. the bridge connections. These bridges are, then, the candidates to be removed in order to effectively detach the communities of a network. For example, in Fig 1 we depict one possible community structure for the Western United States power grid, illustrating the weak connections among clusters that otherwise are densely connected internally. Fig 1A uses nodes to represent a generator, a transformer, or a substation, while edges represent a power supply line. Different colors are used to identify the modules in which that network can be partitioned. In Fig 1B each module is represented by a colored node and edges are shown whenever there is a connection between nodes in different modules (irrespective of how many edges exists). Fig 1C shows the detailed connection between two selected communities displaying all the links among them.

The work is organized as follows: first we address some generalities about attacking complex networks, next we describe our proposed method of attack; then, the results of the procedure applied to ten real networks are reported, and after that conclusions are summarized.

## Network Attack

In general, we can fragment a network by removing either nodes or edges. Removing nodes has an advantage over deleting edges since the removal of a vertex always results in the deletion of all edges attached to it. However, depending on the real system studied, vertex or edge attack may not make sense. For instance, in the case of a road network one may envisage blocking the traffic between two cities, while removing a node would mean to erase an entire village. On the other hand, in biological systems node deletion makes sense since individual metabolites are susceptible to be removed. Later, we will present results regarding both approaches, but from now on, except when explicitly denoted, we will not make such distinction.

A theoretical way of getting the ordered list of targeted nodes to be removed would be by brute force: try all the possible lists until finding the one that reduces the network to a desired size with the minimum number of node deletions. However, this is unfeasible because it means checking *N*! possible lists, which is computational prohibited for any network with *N* ⩾ 12. On the other hand, the simplest but not efficient strategy is the random selection of nodes. This generally results in approximate linear degradation of the network, consequently the atomization of the network is a very slow process in this case. A more efficient and doable way of attacking a graph consists in the deletion of vertices in order of their importance in the structural functioning of the network. In this sense, traditional attacks focus on sorting nodes in decreasing order of some centrality index —the so called Centrality-Based Attack (CBA), which performs much better than random attacks [25–30].

Even though most attack methods focus on centrality ranking, real networks tend to group into sparsely connected clusters. In this sense, the modularity of a partition of an unweighted network can be defined as the density of links inside communities as compared to links between communities [5], as follows:

$$\begin{array}{c} Q=\frac{1}{2m}\sum_{i,j}[A_{ij}-\frac{k_{i}k_{j}}{2m}]\delta \left(c_{i},c_{j}\right)\; \end{array}$$ (1)

where *A*_*ij*_ is the adjacency matrix (taking the value 1 when there is a link between nodes *i* and *j*, 0 otherwise), *k*_*i*_ is the vertex degree of node *i* and *c*_*i*_ represents the community to which this node belongs. The *δ*-function *δ*(*u*, *v*) is 1 if *u* = *v*, 0 otherwise and *m* is the total number of edges. Thus *Q* is a scalar value between -1 and 1 that measures the modularity degree of a network. In other words it gives the actual fraction of the edges inside a community above the expected value of them. The behavior of *Q* is illustrated by S1 Fig which shows the high correlation between the modularity and the fraction of intercommunity edges. In this sense, the removal of few bridging structures in highly modular networks should be able to detach large chunks of densely connected nodes, leading to “fast” fragmentation of complex networks as we shall see in the next section. The term “fast” is used here to refer to a steep response of the network to the removal of nodes, i.e. when a small fraction of nodes is removed, a large fraction of the network is disconnected.

### Module-based attack

The structural importance of a node depends both on local and non-local measures. Hence, in the scope of the method proposed in this paper, centrality and community detection are the topics that we address to characterize and sort nodes in order to develop the attack. As pointed out in the works by Iyer *et al*. [25] and Holme *et al*. [35] nodes with high betweenness and high degree are usually strongly correlated and both attacks have similar efficiency. Besides, the mentioned work by Iyer shows that for real networks betweenness-based methods are in general the most efficient. Thence, from now on we take the betweenness centrality attack as our reference or null method.

Likewise, vertices connecting different communities generally have high betweenness centrality since many shortest paths pass through them. On the other hand, as fewer connections are expected among communities, the nodes that connect them are not necessarily the ones with highest degree. Therefore, in order to detach communities in a more efficient way, we propose a Module-Based Attack (MBA) that loosely resembles the original idea of weak ties proposed by Granovetter [36] for social networks and later developed in the framework of topological communities by De Meo, Ferrara *et al*. [37].

The MBA procedure consists of the following steps:

1. Extract communities using a heuristic detection algorithm (see S1 Text for details).
2. Choose either to attack nodes or edges.
3. Make a list with the nodes (or edges) that participate in intercommunity connections.
4. Sort the list according to (node or edge) betweenness centrality in descending order.
5. Delete nodes (or edges) one by one, starting from the first in the list.
6. While focusing on node removal, once a node from a link between two communities is deleted, its counterpart is skipped from the list (there is no need to remove it), unless it also participates in other intercommunity connections.
7. The attack is always restricted to the largest connected component of the network. In other words, if at some point the next node (edge) in the list does not belong to the remaining largest connected component that node (edge) is skipped.

Notice that the list of nodes to be deleted is obtained only once, before the attacking procedure begins, in what is called simultaneous attack. Sequential attacks (or cascading attacks) [15] are in general more effective because measurements are updated after each deletion. This means that the community detection and betweenness measurement steps have to be rerun after each node (edge) removal. This implies a multiplication of the computation time by the number of nodes to be deleted, making the sequential attack unpractical for large real networks. Besides, due to the recalculation of every topological characteristic after each deletion, we expect all methods of attack to produce more damage per step [25]. Therefore, we would expect the differences between MBA and CBA procedures to decrease (preliminary tests made by us in some cases support such claim). It also should be noted that simultaneous attacks exploit structural weaknesses of networks, which is the main topic of study in this work, while sequential attacks are more related to dynamical properties of complex networks. Therefore, although in this contribution we focus only on simultaneous attacks, the effects of MBA on sequential attacks is an important issue that should be addressed in future works.

## Results

With the aim to demonstrate the validity of the MBA method we apply it to ten real networks with different topological structures and considering all of them as undirected graphs without multiple edges or loops. In order to quantify the effect of the attacks on the networks [38], we define G as an initial network of size *N*, and $G_{\rho }$ as the network that results after the removal of a fraction *ρ* of vertices. Then we denote by $L_{\rho }$ the largest connected component of $G_{\rho }$, whose size is indicated by $N_{L}$. We define the order parameter $\sigma \left(\rho \right)=\frac{N_{L}}{N}$ which allows us to quantify the response of a network to an attack, measured by the relative size of the remainder network as a function of the fraction of nodes (or edges) deleted. In this way, with some method as a null reference, the efficiency gain is defined pointwise for each value of *ρ* as:

$$\begin{array}{c} \gamma \left(\rho \right)=\frac{\sigma_{null}\left(\rho \right)}{\sigma (\rho )} \end{array}$$ (2)

This quantity increases as the attack method becomes more efficient than the reference one. The example networks we have chosen to study are of three types: infrastructural (US power grid, Euro road, Open flights and US airports) [39–46], biological (Yeast protein, C *elegans* and H *pylori*) [47–49] and social (Facebook, Google+ and Twitter) [50–53]. In the Euro road network, nodes represent European cities and edges represent roads. Power grid stands for the electrical power grid of the Western States of the United States of America. An edge represents a power supply line and a node is either a generator, a transformer, or a substation. The Yeast Protein interaction network is the same as in [47]. In the metabolic network of the roundworm *Caenorhabditis elegans* nodes are metabolites (e.g., proteins) and edges are interactions between them. The *Helicobacter pylori* is the same protein-protein interaction map as in [48]. In the Facebook user-user friendship network (NIPS) nodes represent users and edges represent friendship. Similarly, in the Google+ network, an edge means that one user has the other user in her/his circles, while in the Twitter network an edge indicates that both users follow each other. The topological relevant information about the ten networks is presented in Table 1.

**Table 1:** Topological data for the ten real networks studied. size of the networks (*N*), number of edges (*E*), mean degree (〈*k*〉), modularity (*Q*), relative size of the largest community ($N_{mod}^{max}$), fraction of edges linking distinct communities (*E*_*inter*_), and the overall efficiency gain of the MBA method (*η*, see Eq (3) for definition). For the four parameters related with community detection we display the values corresponding to the most efficient case among ten seeds of infomap (I) and ten seeds of *Louvain* (L). These data is presented for node and edge attacks.

					Node Attack				Edge Attack				
**Network**	*N*	*E*	〈*k*〉	*Q*	$N_{mod}^{max}$	*E*_*inter*_	*η*		*Q*	$N_{mod}^{max}$	*E*_*inter*_	*η*	
Facebook	2888	2981	2.06	0.81	0.262	0.012	4.19	(L)	0.81	0.262	0.012	4.19	(L)
Twitter	23370	32831	2.81	0.82	0.018	0.169	38.44	(I)	0.83	0.018	0.168	38.30	(I)
Google Plus	23628	39194	3.32	0.69	0.070	0.279	22.80	(I)	0.69	0.070	0.279	22.80	(I)
US power grid	4941	6594	2.67	0.94	0.049	0.033	111.02	(L)	0.82	0.007	0.178	72.92	(I)
Euro roads	1174	1417	2.41	0.79	0.016	0.203	108.40	(I)	0.79	0.014	0.198	95.16	(I)
Open flights	2939	15677	10.67	0.65	0.184	0.142	8.30	(L)	0.65	0.182	0.139	8.14	(L)
US airports	1574	17215	21.87	0.35	0.296	0.363	4.16	(L)	0.34	0.267	0.341	4.10	(L)
Yeast Protein	1846	2203	2.39	0.77	0.025	0.223	36.14	(I)	0.77	0.025	0.220	35.14	(I)
H *pylori*	724	1403	3.88	0.54	0.124	0.364	19.59	(L)	0.49	0.047	0.485	14.35	(I)
C *elegans*	453	2025	8.94	0.43	0.163	0.423	12.04	(L)	0.43	0.163	0.423	12.04	(L)

**Fig 2:**
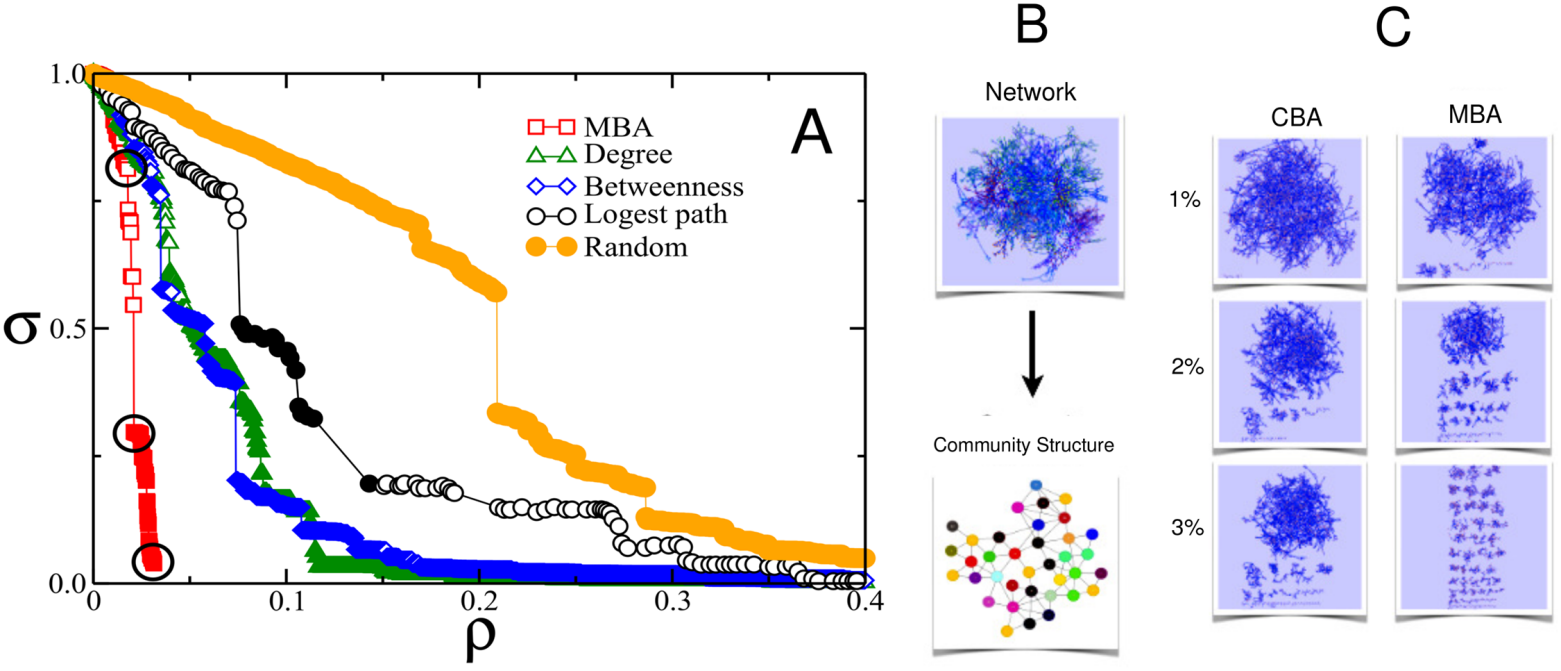
Comparison between the effect of betweenness-based attack, degree-based attack, longest path attack, random attack, and module-based attack for the Western US power grid network. (A) Size of the biggest connected component in terms of the initial size, *σ*, as function of fraction of removed nodes, *ρ*. (B) Network and modular representations of US power grid. (C) Snapshots of the node-representation of the US power grid when 1%, 2% and 3% of nodes are removed using CBA and MBA methods.

From this point on, it was chosen the data corresponding to the most efficient module-based attack over ten seeds of Infomap [32] and ten seeds of *Louvain* [33] community detection algorithms as detailed at S1 Text. Before presenting the results, we illustrate on the attack procedure with a case where the MBA performance is remarkably better than previous and well accepted attacking prescriptions. That example is the power grid of Western USA. Fig 2A summarizes the result of our method of attack as compared to betweenness centrality attack, degree centrality attack, and longest pathway attack [26] for the power grid system. It can also be seen in the figure, the node and modular representations of the US power grid network (Fig 2B) and the snapshots of the network when 1%, 2%, and 3% of nodes are removed by betweenness centrality attack and by the module-based method (Fig 2C). Noteworthy, the present method breaks the original network of 4941 nodes in many fragments smaller than 210 nodes (≈ 4% of the original size) by removing mere 142 nodes (less than 3%) identified by the procedure. By comparison, in any degree or centrality-based procedure, deleting the same amount of nodes, removes only 18% of the original network, *i.e*. more than 4000 nodes continue to be connected after that. Such extreme atomization of the network is well evident in the last snapshot of Fig 2C. Besides, it is quite clear that the community structure of this network is far from trivial (Fig 2B).

**Fig 3:**
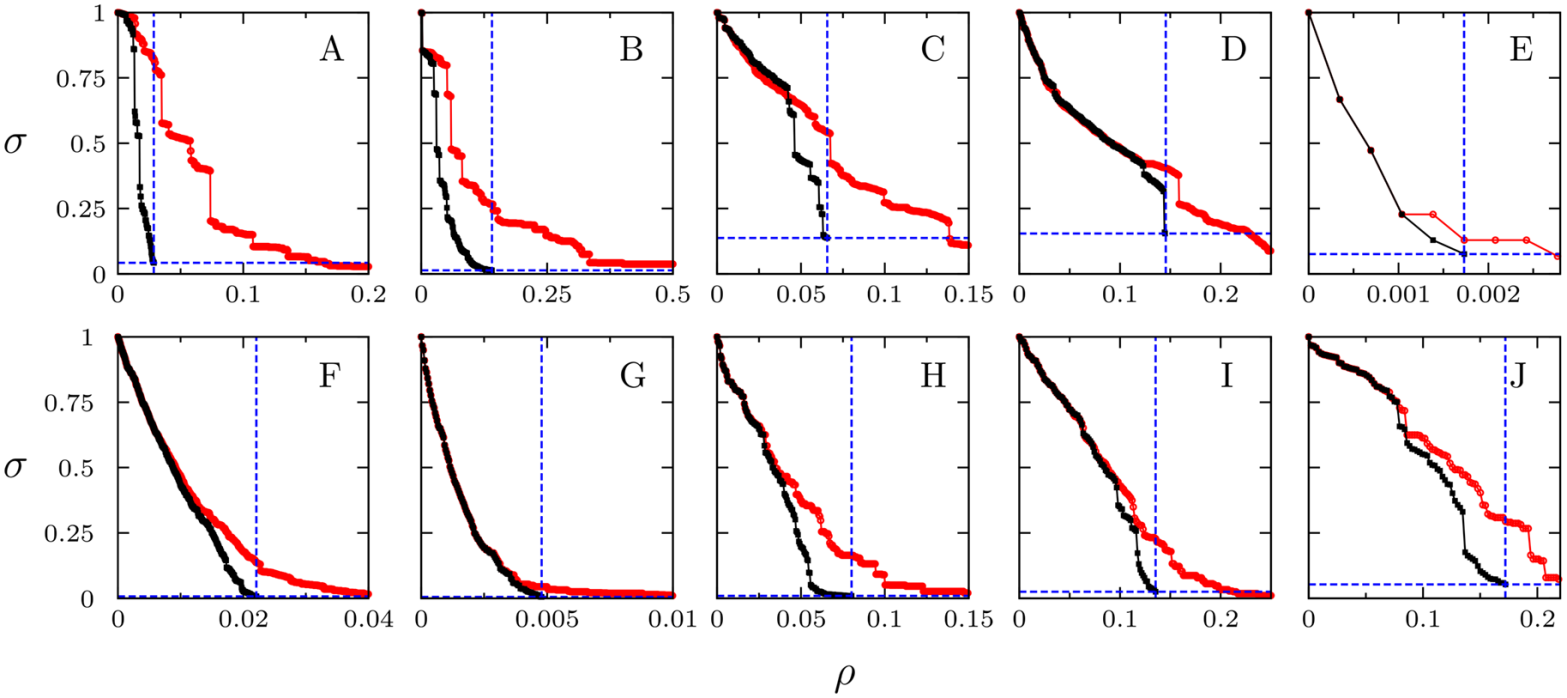
Size of the biggest connected component in terms of the initial size, *σ*, as function of fraction of removed nodes, *ρ*. Vertex Module-based-attack (black squares), betweenness-based attack (red circles). (A) Western US power grid. (B) Euro Road. (C) Open flights. (D) US airports. (E) Facebook. (F) Twitter. (G) Google Plus. (H) Yeast protein. (I) H *pylori*. (J) C *elegans*. The intersection of the dashed blue lines corresponds to the point (*σ*_*e*_, *ρ*_*e*_) of maximum damage on the network using MBA. Network data details are given in Table 1.

**Fig 4:**
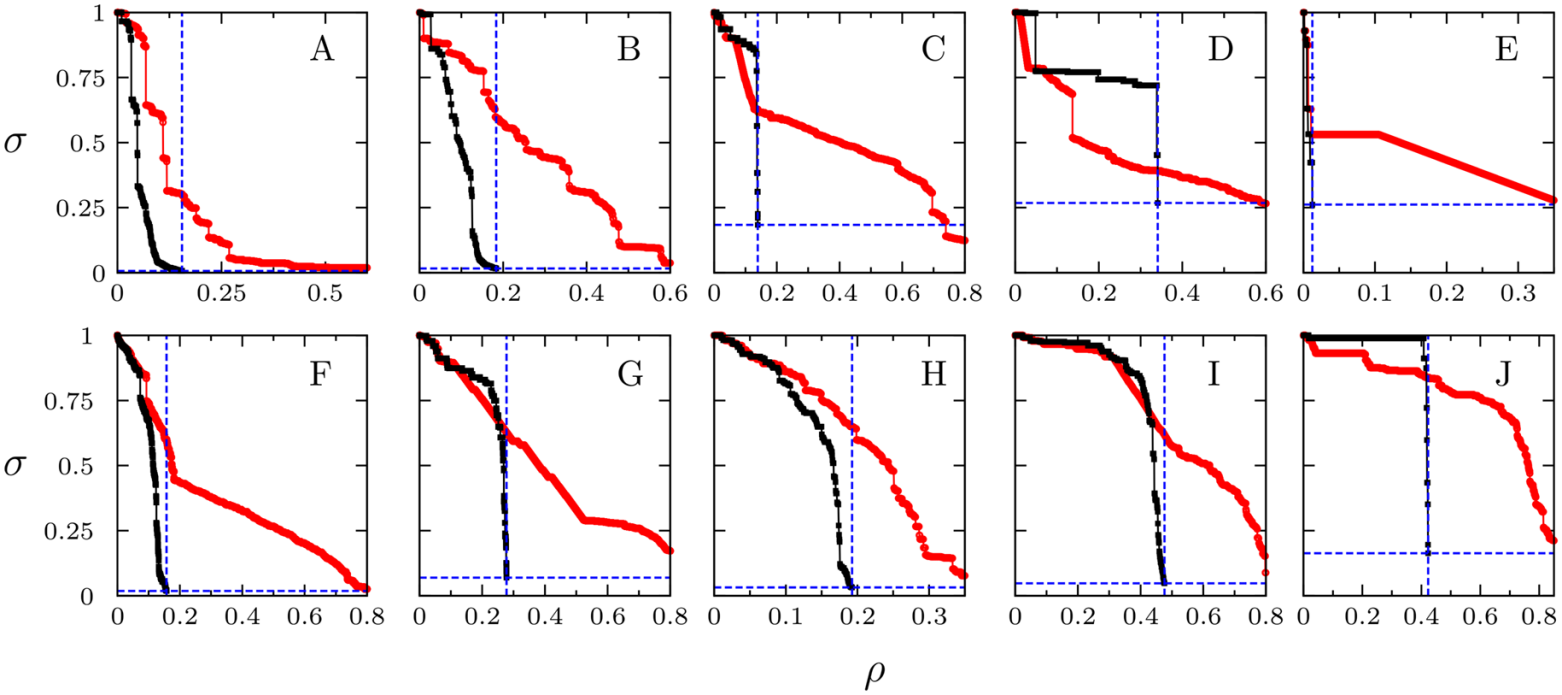
Size of the biggest connected component in terms of the initial size, *σ*, as function of fraction of removed edges, *ρ*. Edges Module-based-attack (black squares), betweenness-based attack (red circles). (A) Western US power grid. (B) Euro road. (C) Open flights. (D) US airports. (E) Facebook. (F) Twitter. (G) Google Plus. (H) Yeast protein. (I) H *pylori*. (J) C *elegans*. The intersection of the dashed blue lines corresponds to the point (*σ*_*e*_, *ρ*_*e*_) of maximum damage on the network using MBA. Network data details are given in Table 1.

Fig 3 displays the results of the vertex MBA applied to the ten networks. Simulations show that vertex MBA always outperforms the traditional betweenness attack. Initially both methods are similar but, as bridges are deleted, whole communities start to detach from the core of the graph, resulting in large atomization of the network and hence in an abrupt decrease of *σ* (meaning an abrupt increase of the efficiency gain *γ*). Results for the same networks, but in the edge MBA procedure, are shown in Fig 4. In this case, as we erase solely edges connecting modules, the initial phase of the attacks is less efficient than CBA attacks for some networks. In these cases we observe a plateau in *σ* before whole modules are effectively detached. After that point is reached, *σ* decreases abruptly, relatively large communities are detached extremely fast, and the whole network falls apart. In both node and edge MBA procedures, attacks stop when the list of nodes or edges is exhausted, *i.e*. at the point when *σ* reaches the minimal ending value *σ*_*e*_. In the edge removal case $\sigma_{e}=N_{mod}^{max}$, where $N_{mod}^{max}$ is the ratio between the largest community and network sizes. On the other hand, the final or ending fraction of edges removed is *ρ*_*e*_ = *E*_*inter*_, where *E*_*inter*_ represents the ratio between the number of edges connecting modules and the total number of edges (see Table 1). In the node removal case $N_{mod}^{max}$ only represent an upper limit for the ending value of *σ*, because additional nodes are detached as a side effect of the procedure, breaking the internal structure of communities —*σ*_*e*_ is in general well below that limit. Besides, in this case *ρ*_*e*_ is far below *E*_*inter*_, because at each node deletion all its edges are removed.

Summarizing the point (*ρ*_*e*_, *σ*_*e*_) (the intersection of the blue dashed lines in Figs 3 and 4), which depends on the particular modular structure of each network, marks where all communities are detached with no targeted node or edge left in the remaining clusters. We can safely say that the network stops functioning as a whole at this point —for instance, information would be stacked within the communities and these structures would not be able to communicate with each other.

**Fig 5:**
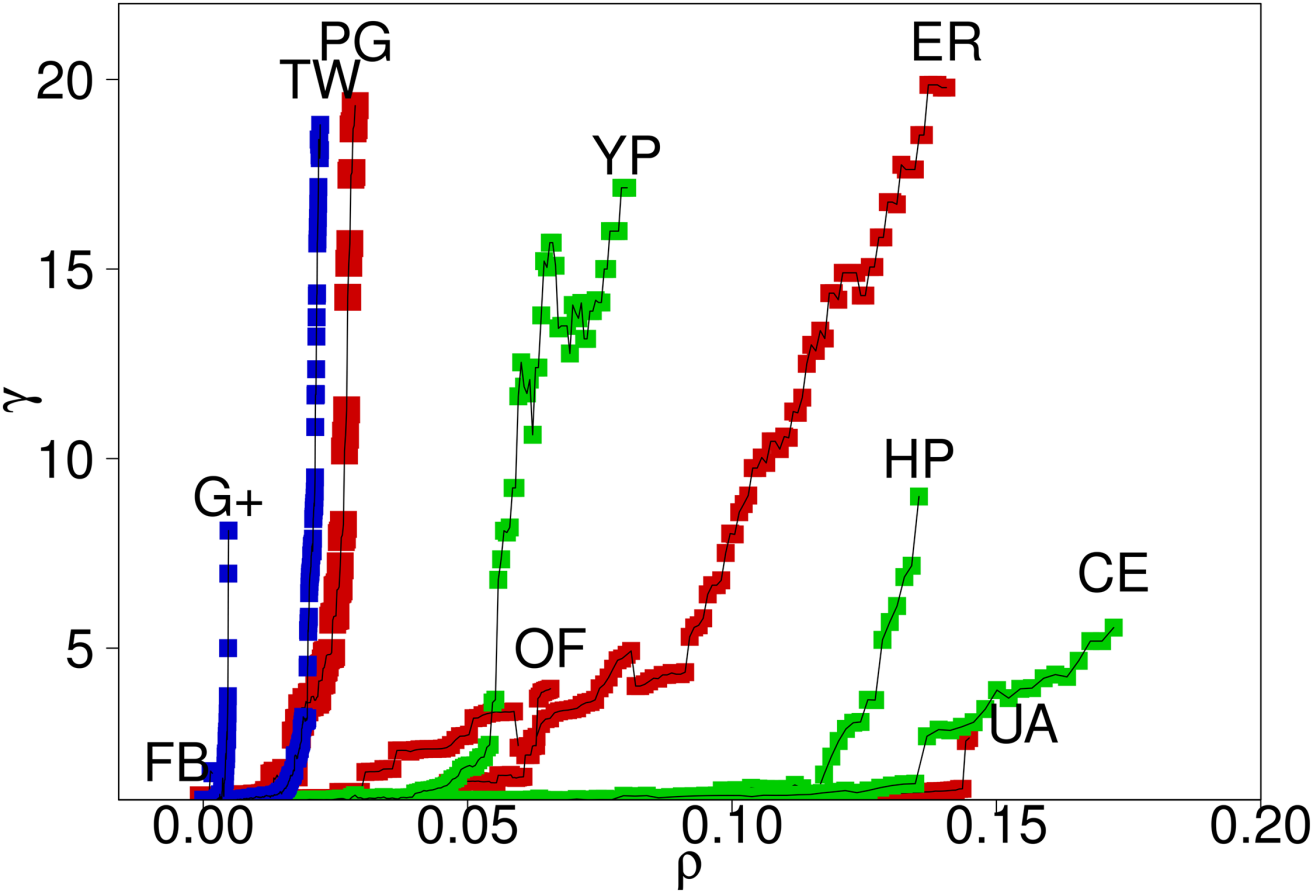
Efficiency gain of vertex MBA, compared to vertex CBA (*γ* = *σ*_*null*_/*σ*), as a function of the fraction of removed nodes, *ρ*. The network code is Facebook (FB), Twitter (TW), Google Plus (G+), US power grid (PG), Euro road (ER), Open flights (OF), US airports (UA), Yeast protein (YP), H *pylori* (HP), and C *elegans* (CE). Infrastructural networks are colored red, biological are colored green, and social are colored blue.

The results presented in Fig 3 can be summarized by means of the relation between *ρ* and *γ* (the efficiency gain of MBA compared to CBA), as displayed in Fig 5 for all the networks. Notoriously, this figure shows that the efficiency is more than doubled for most networks with less than 7% of nodes removed. The most outstanding case is the US power grid with almost 20 times of gain with approximately 3% of nodes removed. Even in the worst cases (H *pylori*, C *elegans*, and US airports) we obtain efficiency gains from 3 to 8 times (relative to CBA method) for 14%-16% of vertices deleted. For instance, the less damaged network in both cases is the US airport network with a final remaining connected fragment of 10% in the node MBA case and of 25% in the edge MBA case. Another feature that emerges from Fig 5 is the clear existence of a “threshold” for the efficiency gain, i.e. a value of *ρ* at which the present procedure clearly departs from the betweenness centrality-based attack.

The overall final performance of the MBA procedure, relative to CBA, may be measured by how fast our method reaches the ending point in comparison with the null CBA method, so we define the overall efficiency gain as:

$$\begin{array}{c} \eta =\gamma \left(\rho_{e}\right)\times \frac{\rho_{null}\left(\sigma_{e}\right)}{\rho_{e}} \end{array}$$ (3)

**Fig 6:**
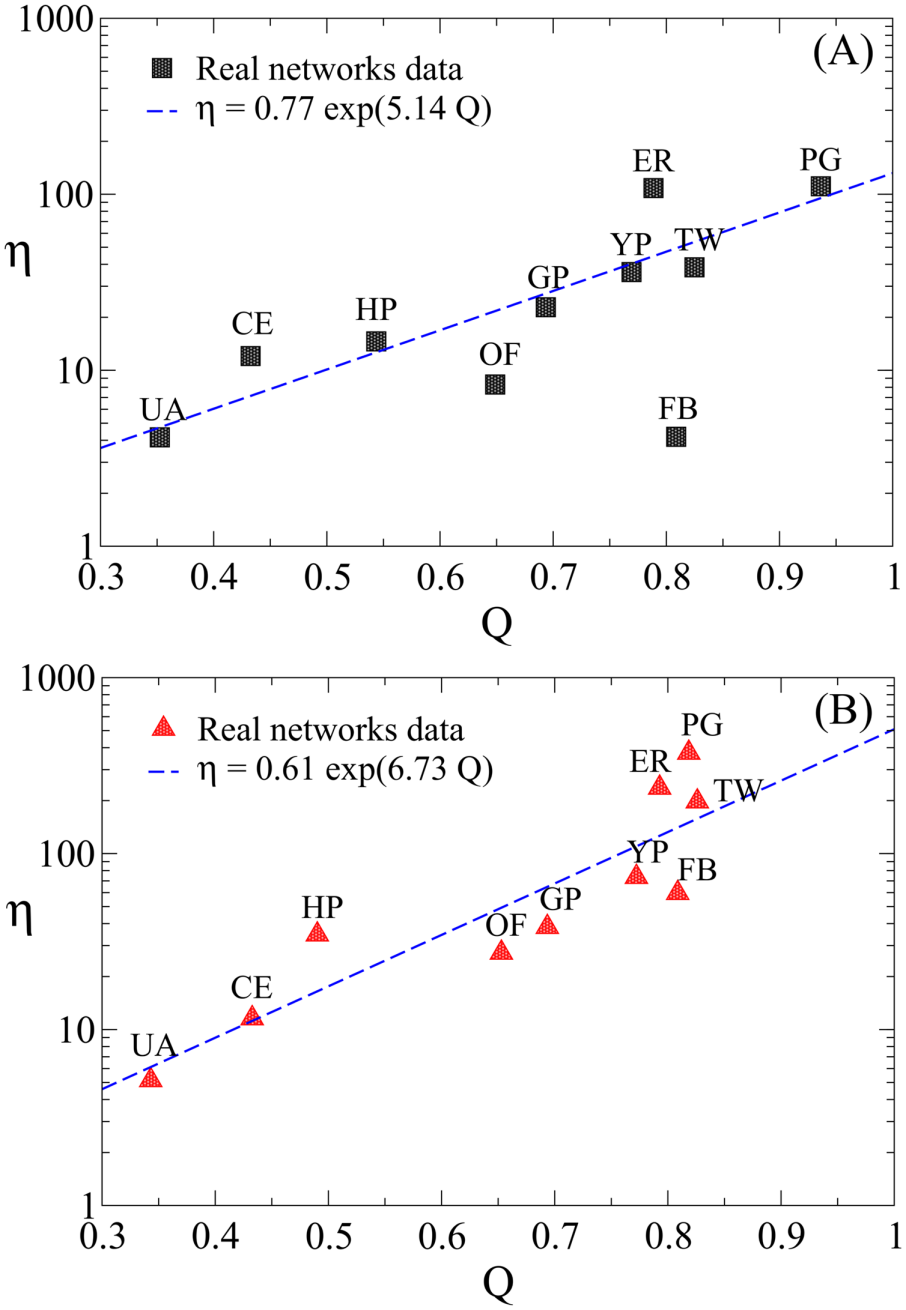
Overall efficiency gain (*η*) of the MBA method relative to the CBA method as function of modularity, *Q*, for nodes and edges removal. The vertical axis is in logarithmic scale and the horizontal axis is linear. The networks attacked are Facebook (FB), Twitter (TW), Google Plus (G+), US power grid (PG), Euro road (ER), Open flights (OF), US airports (UA), Yeast protein (YP), H *pylori* (HP), and C *elegans* (CE).

In Fig 6 we show the results for *η* as a function of *Q* for both type of attacks, vertex and edge MBA, applied to the ten networks. It is quite evident in those figures the existence of a high correlation between *η* and *Q*. Indeed, in both cases, *η* increases steeply with *Q*. This is a highly desirable feature although not easily foreseen. A linear or even a quadratic relation between the overall gain in efficiency *η* and the modularity *Q* would have been easily explained through S1 Fig, which shows a quadratic (inverse) relation between interedges fraction and modularity. However, the gain observed in Fig 6 goes far beyond that expectation. Such a remarkable outcome of *η* and specially its dependence with *Q* may be ascribed to more than one feature of the attack method proposed in this paper. In particular, steps 6 and 7 of our method have a good part of the merit in increasing *η*, by skipping unnecessary nodes from the list during the removal procedure (step 6 only considers inter-modules links, which is exclusive of conducting a module-based attack), and by focusing the attack only in the remaining largest component (step 7 skips nodes from the original list, whose removal at that point would not affect the largest connected component). These two features make a significant reduction on the list of targeted nodes, increasing the efficiency of the method (Fig 2C illustrates on this point for the US power grid example). Yet, there is a particular case which is clearly outside the curve, the Facebook subnetwork. This network is composed of a relative small number of well identified modules (see S3 Fig), all of them organized around highly connected and highly central nodes which happen to be the bridges among communities. Indeed, we can clearly see in Fig 3E that the three first targeted nodes in MBA and CBA are the same nodes; by removing these three nodes the network is reduced to 25% of its original size with no relative gain of the MBA at that point. It can be appreciated how peculiar is this case noting that the whole MBA process ends with just five nodes removed and a final size of ≈6% of the original size. It takes three more nodes for the CBA to reach the same point. Thus, almost any kind of attack, being module-based or centrality-based, has similar effect, even when this network shows a high modularity.

## Discussion

In this work, we have presented a module-based attack method which consists of extracting communities of a given network, then erasing only the nodes that connect distinct modules ordered by betweenness centrality. Computational simulations on many real networks show that the MBA method is more efficient in atomizing networks than traditional procedures based on centrality criteria. Henceforth, one may say that the most connected vertex or the nodes that have the highest value of betweenness centrality are not necessarily the most important for the network survival. Nodes linking distinct communities are structurally more important and crucial for the cohesion of the network than hubs or highly central nodes. If we attack these nodes or its edges, the damage produced to the network is mostly greater than using traditional methods by eliminating the same amount of structures.

The aim of applying the present module-based attack to a given network is to unveil its structural vulnerability, measuring how fast one can attain the regime where the network’s communities are all disconnected. Hence, we propose to characterize the modular vulnerability of complex networks precisely by how fast the ending point *ρ*_*e*_ (where all modules are disconnected) is reached. Outstandingly, the present work shows that the overall gain in efficiency *η* increases quickly with the modularity *Q* of the network, *i.e*. the higher the modularity, the more fragile the network is.

Regarding community detection, the resolution limit of modularity-based algorithms is a topic of debate. However, in connection with the attack method proposed here, it is not highly relevant. The scope of the damage that one can infringe upon a network is related to the number and size of the modules that can be drawn from it. For instance, when large modules are detected, it means the network is decomposed in a few modules, which is good because a large part of the network is disconnected when a module is detached from others. The drawback is that the last module could be still large compared to the original network, as in the US airport network in which the final largest connected component is still of 10% or 25% of the original network, for vertex and edge MBA attacks respectively. On the other hand, a decomposition into many small communities has the advantage of ending with a highly fragmented network, but at the expense of taking more steps than in the other scenario. Therefore, the optimum situation is somehow in the middle, a compromise solution in terms of the average module size and the network size, *i.e*. a biggest module of let say 5% of the unperturbed network.

The identification of communities from the networks, by using the module detection algorithms, is the essential ingredient of our method. And independently of the particular algorithm used to identify the communities, the presented module-based attack method performs always better than traditional methods in fragmenting real networks. Therefore, although these topological modules have no direct relation to real communities, they can eventually disclose some relevant information about the structural functionality of these complex networks.

As a final remark, we want to emphasize the potentiality of the present module-based method in performing attacks on real systems such as disease propagation and terrorists or criminal networks.

## Supporting Information

S1 Fig Modularity and the fraction of bridging links. As a preliminary test of our method we show the relation between the fraction of nodes that connect different modules, *E*_*int*_, and the modularity, *Q*. The data correspond to the ten real networks studied in this work: Facebook (FB), Twitter (TW), Google Plus (G+), US power grid (PG), Euro roads (ER), Open flights (OF), US airports (UA), Yeast protein (YP), H *pylori* (HP) and C *elegans* (CE). As expected, we observe a high (negative) correlation between *E*_*int*_ and *Q*, which is precisely the desired feature that makes the method potentially well posed. The community extraction were performed using either *Louvain* or Infomap methods as detailed in Table 1.
(EPS)

S1 Text Concerning the choice of the community detection algorithm. (PDF)

S2 Fig Sensibility of MBA to different community detection algorithms. We show here the results of different runs of module-based attacks on the US power grid network after community extraction using *Louvain* and Infomap methods. For each algorithm ten different independent realization were acquired.
(EPS)

S3 Fig Networks with high modularity but internally weak. S3 Fig shows the community structure of the Facebook subgraph network. The structure is quite simple, with most of the bridging nodes corresponding to the ones with higher degree. Besides, the internal structure of modules are extremely weak with almost all nodes connected to few vertices or even to only one central node.
(TIFF)

## Abbreviations

CBA: centrality-based attacks
MBA: module-based attacks
