# Supplementary material for: Fast Fragmentation of Networks Using Module-Based Attacks
Source: PLoS One. 2015 Nov 16;10(11):e0142824. doi: 10.1371/journal.pone.0142824 (PMC4646680; doi:10.1371/journal.pone.0142824)
Supplement: S1 Text — (PDF) [file pone.0142824.s002.pdf]

Supporting Information - S1 Text
Fast Fragmentation of Networks using Module-Based Attacks

Bruno Requião da Cunha[1,2], Juan Carlos González-Avella[1,3], Sebastián Gonçalves[1,‡,*],

1 Instituto de Física, Universidade Federal do Rio Grande do Sul, Porto Alegre, RS, Brazil
2 Departamento de Polícia Federal, Brazil
3 Departmento de Física, Pontificia Universidade Católica, Rio de Janeiro, RJ, Brazil

‡Av. Bento Gonçalves 9500 - Caixa Postal 15051 - 91501-970, Porto Alegre, RS - Brazil
*** sgonc@if.ufrgs.br**

The choice of the community detection algorithm

Well established non-heuristic graph partitioning algorithms such as the ones by modularity maximization, statistical inference, and spectral normalized-cut [1] have all a computational requirement drawback for real networks. Most of them are only feasible for networks composed of a few hundred nodes. Henceforth, for real networks with thousands or millions of nodes we must use heuristic algorithms that are much less expensive computationally. On the other hand, the specific choice of the community detection method would impact the MBA efficiency only if the list of bridging nodes and edges changed significantly. In this sense, as pointed out by Fortunato *et al.* [2], the Infomap method by Rosvall and Bergstrom [3] and the *Louvain* method by Blondel and *et al.* [4] are the best performing community detection algorithms for multiple testing benchmarks.

S2 Fig shows the average attack for one network case (PG), represented with a solid line, surrounded by a gray shadow which represents the variation corresponding to ten individual runs of the *Louvain* method and the corresponding curve for ten runs of the Infomap method. As can be seen, the variation among different instances of the community extraction algorithm is very small compared to the difference between MBA and CBA method. Although there are some differences in the definition of the communities, the behavior of the MBA curves are very similar and the bands of both methods strongly overlap. Therefore both methods of community extraction, Infomap and *Louvain*, are equally suited to be used as part for the attacking method.

References

1. Newman MEJ. Spectral methods for community detection and graph partitioning. Phys Rev E. 2013 Oct;88:042822. Available from: `http://link.aps.org/doi/10.1103/PhysRevE.88.042822`.

2. Lancichinetti A, Fortunato S. Community detection algorithms: A comparative analysis. Phys Rev E. 2009 Nov;80:056117. Available from: `http://link.aps.org/doi/10.1103/PhysRevE.80.056117`.

3. Rosvall M, Axelsson D, Bergstrom CT. The map equation. The European Physical Journal Special Topics. 2009;178(1):13–23. Available from: http://dx.doi.org/10.1140/epjst/e2010-01179-1.

4. Blondel VD, Guillaume JL, Lambiotte R, Lefebvre E. Fast unfolding of communities in large networks. Journal of Statistical Mechanics: Theory and Experiment. 2008;2008(10):P10008. Available from: http://stacks.iop.org/1742-5468/2008/i=10/a=P10008.
