# Supplementary figures and images for: Fast Fragmentation of Networks Using Module-Based Attacks
Source: PLoS One. 2015 Nov 16;10(11):e0142824. doi: 10.1371/journal.pone.0142824 (PMC4646680; doi:10.1371/journal.pone.0142824)

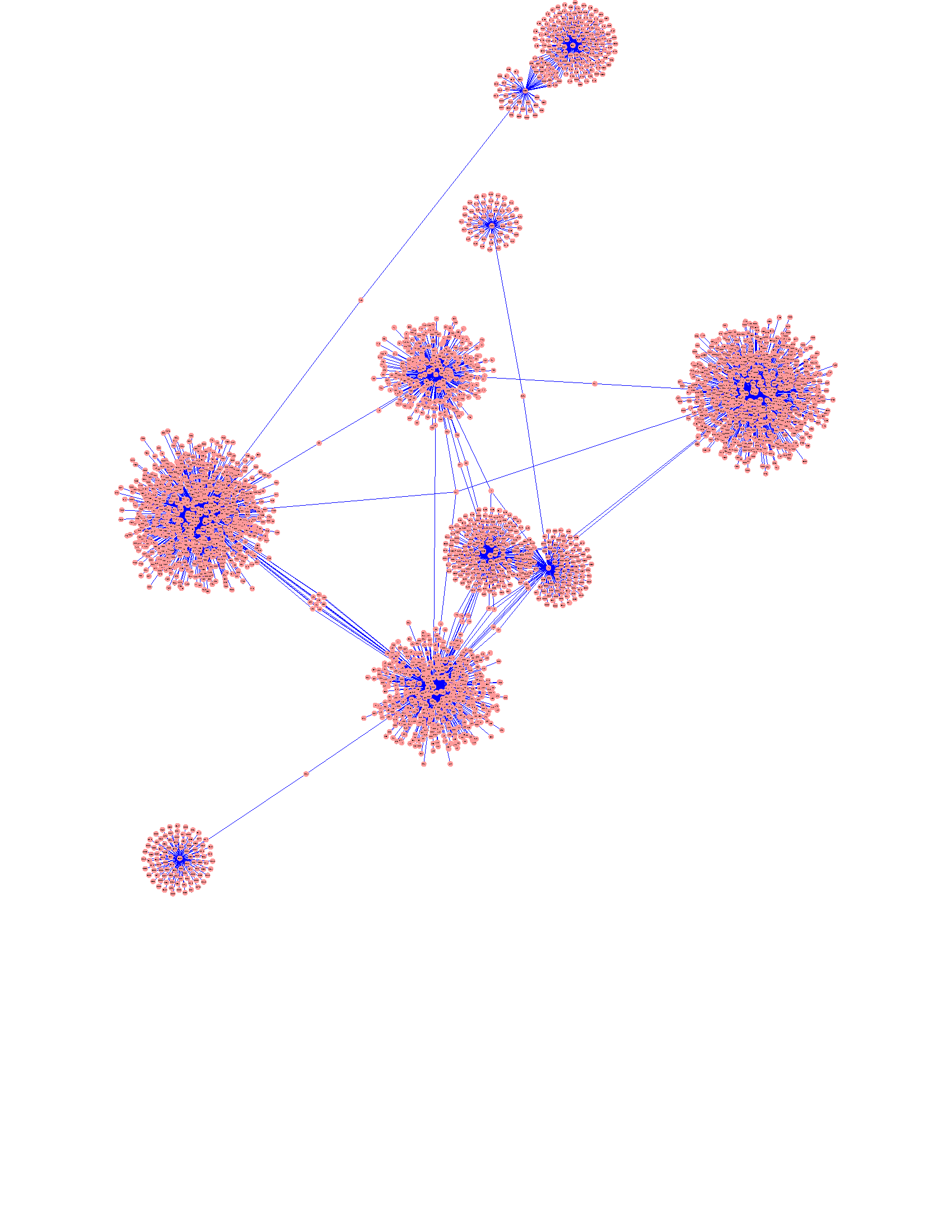

Supplement: S3 Fig — shows the community structure of the Facebook subgraph network. The structure is quite simple, with most of the bridging nodes corresponding to the ones with higher degree. Besides, the internal structure of modules are extremely weak with almost all nodes connected to few vertices or even to only one central node. (TIFF) [file pone.0142824.s004.tiff]
